# Metabolites from the *Dendrobium* Endophyte *Pseudomonas protegens* CM-YJ44 Alleviate Insulin Resistance in HepG2 Cells via the IRS1/PI3K/Akt/GSK3β/GLUT4 Pathway

**DOI:** 10.3390/ph18060817

**Published:** 2025-05-29

**Authors:** Luqi Qin, Yixia Zhou, Bei Fan, Jiahuan Zheng, Rao Diao, Jiameng Liu, Fengzhong Wang

**Affiliations:** 1 Key Laboratory of Agro-Products Quality and Safety Control in Storage and Transport Process, Ministry of Agriculture and Rural Affairs, Institute of Food Science and Technology, Chinese Academy of Agricultural Sciences, No. 2, Yuanmingyuan West Road, Haidian District, Beijing 100193, China; qlq120011@163.com (L.Q.); zyx13967696166@163.com (Y.Z.); zjiah1996@163.com (J.Z.); diaodiao19971126@sina.com (R.D.); 2 National Nanfan Research Institute (Sanya), Chinese Academy of Agricultural Sciences, Sanya 572024, China

**Keywords:** *Dendrobium* endophyte, insulin resistance, PI3K/Akt Pathway, molecular docking

## Abstract

**Background/Objectives:** Endophytes can produce bioactive metabolites similar to their host plants. CM-YJ44 (*Pseudomonas protegens* CHA0, 99.24% similarity), an endophyte from *Dendrobium officinale*, has not yet validated hypoglycemic potential. This study aimed to evaluate its anti-insulin resistance (IR) activity and metabolite profile. **Methods:** The fermentation broth of CM-YJ44 was separated into three fractions (CM-YJ44-1, -2, and -3) using semi-preparative high-performance liquid chromatography (pre-HPLC). An IR HepG2 cell model was constructed to evaluate their glucose uptake capacity. CM-YJ44-3 was further tested for oxidative stress, inflammatory, and insulin signaling pathway activation. Metabolites in CM-YJ44-3 were preliminarily identified using the Q Exactive Focus LC-MS system (QE), and the dendrobine content was quantified by ultra-performance liquid chromatography–tandem mass spectrometry (UPLC-MS/MS). Molecular docking was performed to predict the binding affinities between dendrobine and target proteins. **Results:** Among the three fractions, CM-YJ44-3 significantly reduced nitric oxide (NO) and reactive oxygen species (ROS) levels in IR cells, enhanced glycogen synthesis, upregulated the activities of pyruvate kinase (PK) and hexokinase (HK), and suppressed the expression of inflammatory factors. Its mechanism of action was mainly through activation of the IRS1/PI3K/Akt/GSK3β/GLUT4 signaling pathway. QE analysis preliminarily identified 24 metabolites in CM-YJ44-3. Quantitative analysis by UPLC-MS/MS showed that the dendrobine content was 78.73 ± 4.29 ng/mL. Molecular docking results indicated that dendrobine exhibited binding energies below −5 kcal/mol with multiple target proteins involved in this signaling pathway, suggesting it may be a key bioactive component responsible for the anti-IR effect. **Conclusions:** This study provides the first evidence of hypoglycemic bioactive metabolite production by strain CM-YJ44, indicating its potential as a novel microbial candidate for alleviating IR.

## 1. Introduction

Diabetes has increasingly become a global health issue, and the number of cases is projected to reach 702 million by 2045 according to the International Diabetes Federation [1]. The most common form of diabetes is type 2 diabetes (T2D), characterized by inadequate insulin secretion and IR. IR is defined as a diminished responsiveness of peripheral tissues to insulin signals in muscle, liver, and adipose tissues. It could weaken the ability of insulin to regulate glucose metabolism, including glucose uptake and utilization, lipolysis, and glycogen synthesis [2]. The deterioration of IR impairs the capacity of insulin to modulate glucose metabolism within the body, ultimately resulting in hyperglycemia and subsequent progression to T2DM. Therefore, preventing the state of IR from developing into T2D is considered an important strategy in the treatment of T2D.

The development of IR is a complex process, primarily influenced by genetic factors and the individual’s physiological state. Numerous studies have pointed out that occurrence of IR is mainly associated with insulin signaling blockade, oxidative stress, dysfunction of adipocytes, and dysregulation of glycogen synthesis [3]. As the core of insulin signaling, the phosphorylation of insulin receptor substrate 1 (IRS1) activates downstream effectors in the phosphatidylinositol 3-kinase (PI3K)/protein kinase B (Akt) pathway [4], which is crucial for insulin action. The expression levels of PI3K and Akt are crucial for the regulation of downstream targets, including glycogen synthase kinase 3 beta (GSK3β) and glucose transporters (GLUTs). Specifically, Akt inhibits the activity of GSK3β through phosphorylation, thereby relieving its inhibitory effect on glycogen synthase and promoting glycogen synthesis [4]. In addition, GLUT4, a key glucose transporter in the liver, is mobilized and swiftly translocated from intracellular compartments to the plasma membrane in response to insulin stimulation, allowing glucose to enter target cells along its concentration gradient [5]. This mechanism plays a vital role in blood glucose regulation, with the PI3K/Akt signaling pathway proven to participate in this process [6]. As a result, the PI3K/Akt pathway is regarded as one of the most crucial targets for investigating strategies for improving IR.

Currently, commonly used antidiabetic drugs such as acarbose, metformin (MET), and rosiglitazone are associated with side effects like bloating, diarrhea, and nausea [7,8,9]. Therefore, developing effective treatments that can improve IR with minimal or no adverse effects is of great significance. Natural metabolites derived from plants have been demonstrated to show hypoglycemic effects, indicating that they can be valuable sources to develop antidiabetic drugs [10]. *Dendrobium officinale*, a rare traditional Chinese medicinal herb, is considered to be an edible medicinal plant. The principal bioactive components include dendrobine [11], dendrobium polysaccharides [12], and dendrobium polyphenols [13]. The observed antioxidant, anti-inflammatory, and hypoglycemic activities in *Dendrobium officinale* and its endophytic strains are likely attributable to synergistic interactions among these bioactive components [14]. Current studies have confirmed that endophytes isolated from medicinal plants could produce the same or comparable active metabolites as their hosts [15,16]. Consequently, they can be considered as a promising reservoir of novel natural products for food, pharmaceutical, and agricultural industries.

In our previous study, an endophytic fungus, CM-YJ44, was isolated from the stem of *Dendrobium officinale* [17]. However, the main metabolites produced during its fermentation process remain unclear. Additionally, whether these metabolites possess the same hypoglycemic effects as *Dendrobium officinale*, as well as the specific mechanisms of action, still require further investigation. Therefore, this study aims to evaluate the therapeutic potential of CM-YJ44 in alleviating IR, identify its main metabolite components, and explore the alleviating IR molecular mechanisms. This research may provide new insights into the pharmacological actions of endophytic fungi and their potential applications in developing hypoglycemic drugs and functional foods for blood sugar regulation.

## 2. Results

### 2.1. Establishment of the IR Model

**Figure 1:**
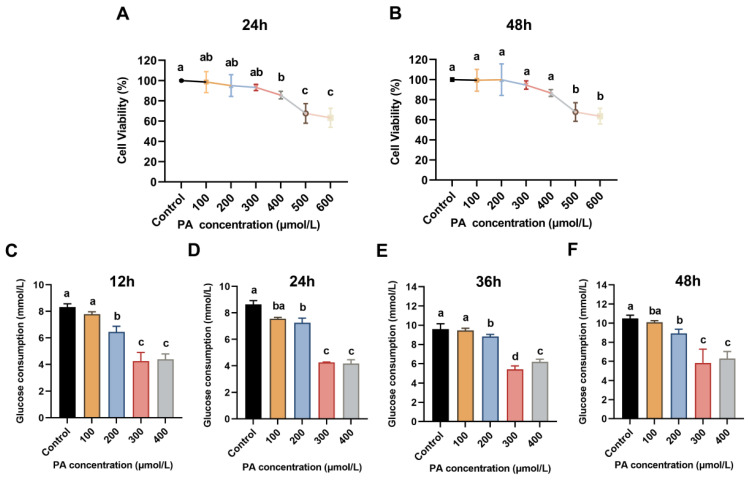
Establishment of the IR HepG2 cell model. Cell viability of HepG2 cells in various concentrations of PA for 24 h (**A**) and 48 h (**B**), respectively. Effect of different concentrations and various treatment times of PA for 12 h (**C**), 24 h (**D**), 36 h (**E**), and 48 h (**F**) on glucose consumption. Data are expressed as the means ± SD (*n* = 3). Different letters indicate significant difference (*p* < 0.05), while the same letters indicate no significant difference.

Generally, under the stimulation of PA at a high level, the number of insulin receptors on the surface of HepG2 cells decreases, which is mainly correlated with the level of PA and stimulation duration [18]. Therefore, HepG2 cells are widely used as a suitable in vitro model for investigating the mechanisms underlying IR and assessing the efficacy of hypoglycemic agents. In this study, to optimize the IR model, different concentrations of PA and induction durations were initially evaluated. As shown in Figure 1A,B, the impact of varying PA concentrations and incubation times on HepG2 cell viability was examined. Compared with the control group, PA at concentrations between 100 and 300 µmol/L did not exhibit any inhibitory effect on cell viability, regardless of whether the induction lasted 24 or 48 h. However, the cell viability was obviously decreased to below 90%, with the concentration of PA exceeding 400 μmol/L.

The screening results for the optimal PA concentration demonstrated a progressive decline in glucose consumption as PA levels increased from 100 to 300 µmol/L (Figure 1C–F), indicating that higher PA concentrations were observed to induce a pronounced IR phenotype in HepG2 cells. However, when the PA concentration reached 400 µmol/L, glucose consumption unexpectedly increased. It was postulated that supraoptimal levels of PA (beyond the optimal induction concentration) could impair cellular homeostasis of HepG2 cells and disrupt cell membrane integrity, potentially facilitating the diffusion of glucose into the cells. Glucose consumption was lowest when the insulin concentration was 300 μmol/L, regardless of whether induced for 12, 24, 36, or 48 h. The largest difference in glucose consumption between the control and model groups was observed at 36 h of induction, with a maximum difference of 4.18 mmol/L. Moreover, this concentration showed no impact on cell viability. Therefore, 300 µmol/L PA and 36 h were selected as the optimal concentration and inducing time to establish the cell model, respectively.

### 2.2. Glucose Consumption of CM-YJ44 Fermentation Broth on the IR HepG2 Cell

**Figure 2:**
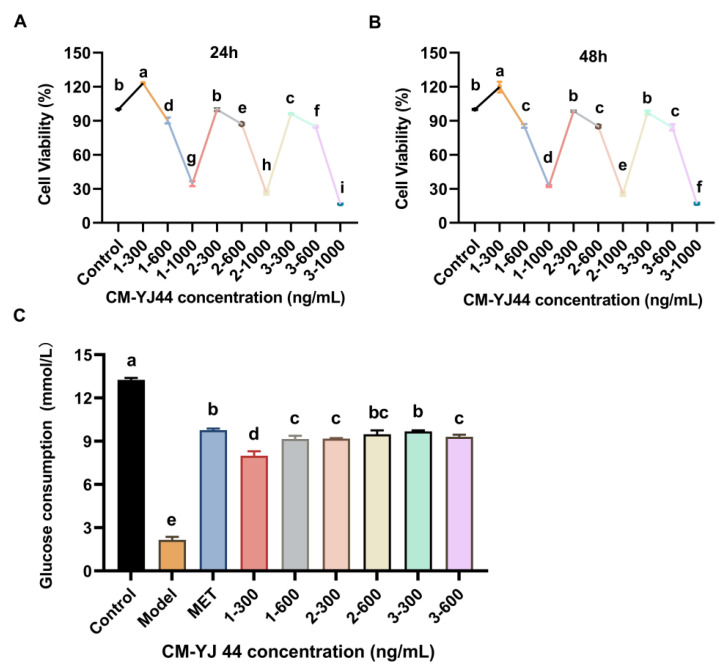
Effects of fermentation broth extracts from different fractions of CM-YJ44 on HepG2 cells. (**A**) Cell viability of HepG2 cells in different fractions and concentrations of fermentation broth extract of CM-YJ44 (100, 200, 300, 400, 500, and 600 ng/mL) for 24 h and (**B**) 48 h. (**C**) Effect of different collection segments and concentrations of fermentation broth extract CM-YJ44 on glucose consumption in the insulin-resistant model. MET was used as the positive control. Data are expressed as the means ± SD (*n* = 3). Different letters indicate significant difference (*p* < 0.05), while same letters indicate no significant difference.

The activities of three fractions of CM-YJ44 fermentation broth on cell viability and glucose consumption in IR cell model were detected separately. As shown in Figure 2A–C, all three fractions of CM-YJ44 at concentrations of 600 and 1000 ng/mL exhibited obvious cytotoxicity, while 300 ng/mL of CM-YJ44 showed no significant effect on cell viability and was considered a safe concentration. In addition, CM-YJ44-1 (fraction 1) at 300 ng/mL had a proliferative effect on cells. Subsequently, the glucose consumption of the three fractions was measured, and the results showed that all the fractions significantly improved glucose consumption compared with the model group, among which the concentration 300 ng/mL of the CM-YJ44-3 (fraction 3) exhibited the most significant influence that the glucose consumption was close to MET group. The above results indicated that CM-YJ44 could improve glucose consumption and alleviate IR in HepG2, with CM-YJ44-3 demonstrating the most prominent activity.

### 2.3. Effects of CM-YJ44-3 on Blood Glucose, Glycogen, HK, and PK in IR Cells

**Figure 3:**
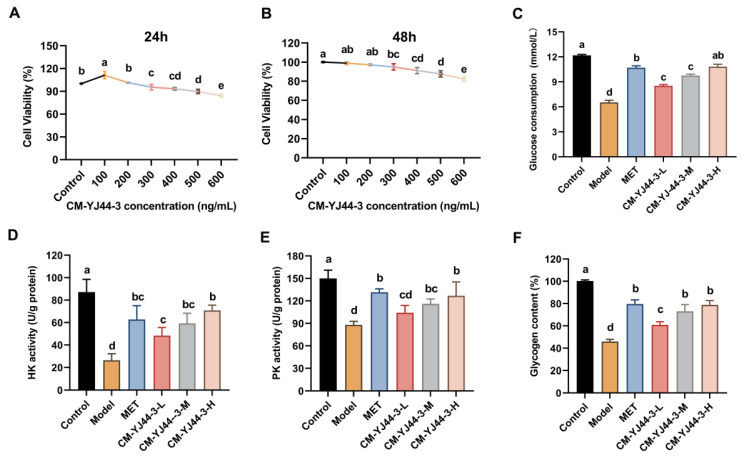
Effect of CM-YJ44-3 on HepG2 cells and effect of CM-YJ44-3 on activities of HK, PK, and glycogen content. (**A**) Cell viability of HepG2 cells in different concentrations of CM-YJ44-3 (100, 200, 300, 400, 500, and 600 ng/mL) for 24 h and (**B**) 48 h. (**C**) Effect of different doses of CM-YJ44-3 on glucose consumption in the insulin-resistant model. Effect of different doses of CM-YJ44-3 on activities of HK (**D**), PK (**E**), and glycogen content (**F**) in the insulin-resistant model. MET was considered the positive control. Data are represented as the means ± SD (*n* = 3). MET was used as the positive control. Data are expressed as the means ± SD (*n* = 3). Different letters indicate significant difference (*p* < 0.05), while same letters indicate no significant difference.

The effects of CM-YJ44-3 on cell viability were detected before the glucose consumption was measured. It was found that CM-YJ44-3 (0–300 ng/mL) treatment for 24 h or 48 h did not affect the viability of HepG2 cells (>80%), while cell viability was significantly reduced at higher doses (after administration of 500 ng/mL HepG2 cells (Figure 3A,B). Thus, CM-YJ44-3 concentration below 300 ng/mL was used in subsequent experiments. Subsequently, the levels of glucose uptake and glucose consumption in cultured cells were measured. Compared with the model group, glucose consumption increased significantly in both the control and treatment groups. After CM-YJ44-3 treatment, glucose consumption was raised dose-dependently, reaching 8.52 ± 0.16 mmol/mL, 9.76 ± 0.18 mmol/mL, and 10.82 ± 0.30 mmol/mL, respectively. Notably, the glucose consumption of the CM-YJ44-3-H group exhibited superior glucose consumption capacity compared to the MET group (10.71 ± 0.22 mmol/mL), approaching the control group (12.19 ± 0.10 mmol/mL).

Insulin regulates blood glucose by stimulating glycogen synthesis in target tissues, such as the muscle and liver, while concurrently suppressing glycogen breakdown [19]. As shown in Figure 3D–F, after treatment with CM-YJ44-3, glycogen content in the CM-YJ44-3-M and CM-YJ44-3-H groups did not show a significant difference compared with the MET group but was significantly higher than that of the model group, with increases of 0.59-fold and 0.72-fold. As the crucial enzymes in glycolysis, HK and PK can regulate the metabolism of glucose. In this study, the activities of HK (26.46 ± 5.75) and PK (88.00 ± 4.71) were significantly suppressed in the model group. Treatment with CM-YJ44-3 effectively reversed this inhibition, increasing HK activity to 48.38 ± 7.28, 59.24 ± 9.15, and 70.88 ± 4.69; and PK activity increasing to 60.86 ± 2.86, 73.10 ± 6.06, and 78.83 ± 3.95 at low, medium, and high concentrations of CM-YJ44-3, respectively.

### 2.4. Effects of CM-YJ44-3 on Generation of ROS and NO

**Figure 4:**
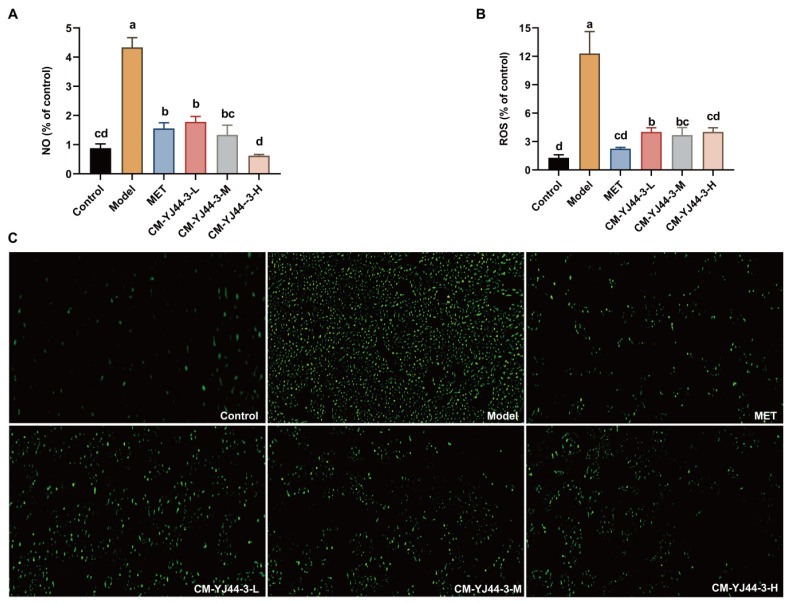
Statistics of ROS and NO expression in CM-YJ-44-3. No expression in insulin resistance model with different dose of CM-YJ44-3 (**A**). Fluorescence image (**C**) (original magnification, ×200; 20× objective and 10× eyepiece) and statistics (**B**) of ROS in insulin resistance model with different doses of CM-YJ44-3. MET was used as the positive control. Data are expressed as the means ± SD (*n* = 3). Different letters indicate significant difference (*p* < 0.05), while same letters indicate no significant difference.

Persistent hyperglycemia induces oxidative stress, subsequently triggering inflammation in peripheral tissues, impairing IR, reducing glucose utilization, and accelerating diabetic complication progression. Elevated levels of ROS are unquestionably a key contributor to the progression of diabetes and its related complications [20]. Considering the critical roles of oxidative stress and inflammation in the pathogenesis of diabetes, the effects of CM-YJ44-3 on ROS, NO, and inflammatory markers in IR cells were assessed. As shown in Figure 4A, NO levels were significantly higher in the IR model group compared with the control group. However, after being treated with CM-YJ44-3, the content of NO was significantly downregulated and close to the MET group. NO would be synthesized in the stress response with ROS generated. In other words, the ROS level was decreased by downregulating NO levels. As expected, ROS levels were elevated in the IR model group, but the overproduction of ROS was effectively suppressed after treatment with CM-YJ44-3 (Figure 4B,C).

### 2.5. Effect of CM-YJ44-3 on PI3K/Akt Signaling Pathway

**Figure 5:**
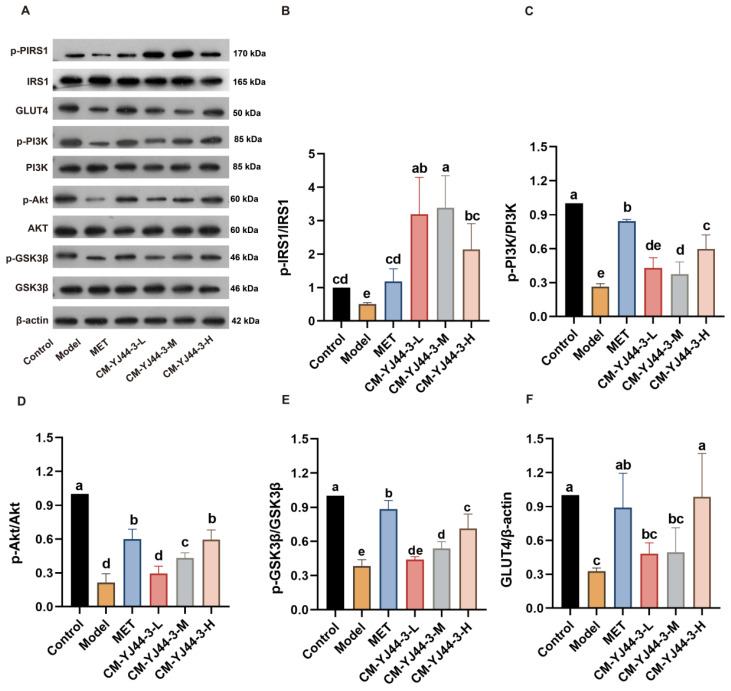
Effect of different doses of CM-YJ44-3 on IRS1/PI3K/Akt/GSK3β/GLUT4 signaling pathway in the IR model. (**A**) Western blots of p-IRS1, IRS1, GLUT4, p-PI3K, PI3K, p-Akt, Akt, P-GSK3β, GSK3β, and β-actin protein expressions. Relative expressions of p-IRS1/IRS1 (**B**), p-PI3K/PI3K (**C**) p-Akt/Akt (**D**), P-GSK3β/GSK3β (**E**), and GLUT4 (**F**). MET was used as the positive control. Data are expressed as the means ± SD (*n* = 3). Different letters indicate significant difference (*p* < 0.05), while same letters indicate no significant difference.

We focused on the IRS1/PI3K/Akt/GSK3β/GLUT4 signaling pathway. According to the Western blot bands of p-IRS1, IRS1, GLUT4, p-PI3K, PI3K, p-Akt, Akt, p-GSK3β, GSK3β, and β-actin (Figure 5A) revealed that the PI3K/Akt axis was activated by CM-YJ44-3 treatment, as expected, which played a crucial role in regulating glucose metabolism.

Figure 5B shows that IRS1 activity was inhibited in the IR model group, but CM-YJ44-3 treatment significantly increased IRS1 phosphorylation. As an upstream component of the PI3K/Akt signaling pathway, IRS1 regulates the expression of PI3K and Akt. The results showed that CM-YJ44-3 effectively reversed the IR-induced reduction in p-Akt and p-PI3K levels (Figure 5C). The PI3K/Akt pathway modulates key downstream proteins involved in insulin signaling, such as GSK3β and GLUT4. Our results showed that CM-YJ44-3-M and CM-YJ44-3-H treatments significantly elevated p-GSK3β expression, with increased of 0.74-fold and 1.5-fold, respectively. Additionally CM-YJ44-3-H treatment notably enhanced GLUT4 expression by 2-fold (Figure 5F). Unfortunately, although CM-YJ44-3-L group could increased the expression levels of p-GSK3β and GLUT4, it did not yet reach a significant difference.

### 2.6. Effect of CM-YJ44-3 on the Levels of Inflammatory Factors

**Figure 6:**
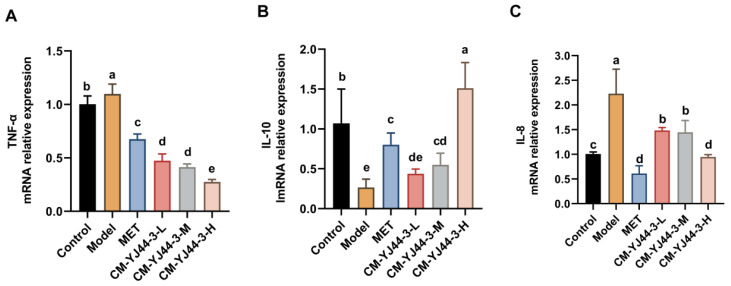
The mRNA levels of TNF-α (**A**), IL-8 (**B**), and IL-10 (**C**) were determined by PCR. The β-actin was used as control. MET was used as the positive control. Data are expressed as the means ± SD (*n* = 3). Different letters indicate significant difference (*p* < 0.05), while same letters indicate no significant difference.

IL-8, IL-10, and TNF-α are typical inflammatory factors, so the mRNA levels of pro-inflammatory factors IL-8, TNF-α, and anti-inflammatory factor IL-10 were detected, respectively (Figure 6). The results revealed a 1.10-fold elevation of TNF-α in the IR model group, contrasted with a marked 2.23-fold upregulation of IL-8 expression compared to the control group. Treatment with CM-YJ44-3 effectively suppressed the expression of these pro-inflammatory markers. Specifically, TNF-α expression decreased to 0.47-fold, 0.41-fold, and 0.27-fold in the low-, medium-, and high-dose CM-YJ44-3 groups, respectively, while the MET group showed a decrease to 0.68-fold. Similarly, IL-8 expression was reduced to 1.48-fold, 1.45-fold, and 0.95-fold in the corresponding CM-YJ44-3 groups, with the high-dose group approaching normal levels. As for IL-10, its expression was significantly downregulated in the IR model group, reaching only 0.27-fold of the control level. CM-YJ44-3 treatment led to a dose-dependent restoration, increasing IL-10 levels to 0.44-fold, 0.55-fold, and 1.51-fold in the low-, medium-, and high-dose groups, respectively. The MET group also showed a moderate increase to 0.80-fold.

These results suggested that CM-YJ44-3 could effectively ameliorate IR-induced inflammation by downregulating pro-inflammatory and upregulating anti-inflammatory cytokine gene expression in a dose-dependent modulation.

### 2.7. Analysis of the Metabolites from CM-YJ44-3

Given the significant hypoglycemic and antioxidant activities exhibited by CM-YJ44-3, comprehensive identification and analysis of its metabolic products were performed. As shown in Table 1, a total of 24 metabolites, including sesquiterpenes, phenolics, and saponins, were preliminarily identified based on qualitative analysis, and their reported origins are also listed (total ion chromatogram shown in Supplementary Figure S2). It was found that many of the metabolites partially overlapped with the secondary metabolites of the host plant, *Dendrobium officinale*, including alkaloids and polyphenols [21], which may be attributed to the specific adaptive mechanisms of the endophytes. Previous studies have proposed that endophytic microorganisms could acquire genetic elements associated with host metabolic pathways through long-term symbiotic relationships, either by metabolic co-evolution or horizontal gene transfer, thereby developing biosynthetic capabilities to produce structurally analogous or functionally comparable compounds [22,23,24]. In addition, to maintain the intrinsic metabolic profile of strain CM-YJ44, the number of subcultures was strictly limited after the isolation, minimizing phenotypic variation caused by prolonged in vitro cultivation. Notably, certain metabolites (such as protopine) have not been previously reported from microbial sources and were detected for the first time in *Pseudomonas*. While structural confirmation of those compounds remains necessary, these findings partially indicated the strain’s capacity to produce structurally identical or related compounds, meriting additional study.

**Figure 7:**
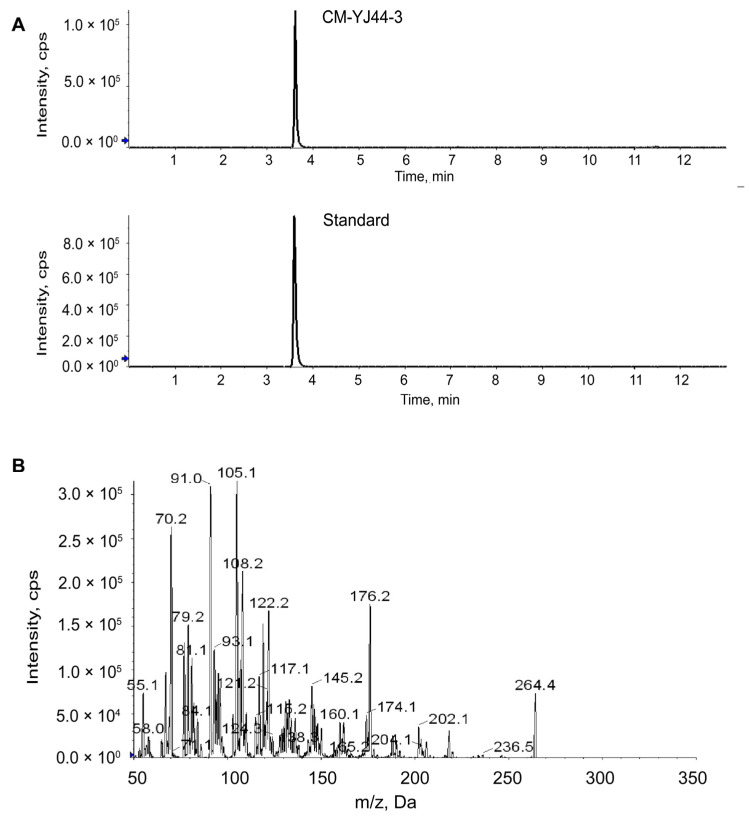
UPLC-MS/MS analysis dendrobine of CM-YJ44-3. (**A**) UPLC-MS/MS chromatogram of dendrobine standard controls and CM-YJ44-3. (**B**) The MS/MS fragmentation spectrum of dendrobine in CM-YJ44-3 sample by UPLC-MS/MS.

Based on the whole-genome sequencing results of CM-YJ44 obtained previously [17], the biosynthetic capacity of this strain for sesquiterpene alkaloids was further confirmed, prompting us to focus on the detection of dendrobine. As shown in Figure 7, based on its retention time, precursor ion consistency, and diagnostic MS/MS fragmentation pattern with the dendrobine standard, dendrobine was preliminarily identified in CM-YJ44-3. As shown in Table 2, dendrobine was quantified at a concentration of 78.73 ± 4.29 ng/mL (chromatogram shown in Supplementary Figure S4).

### 2.8. Molecular Docking

**Figure 8:**
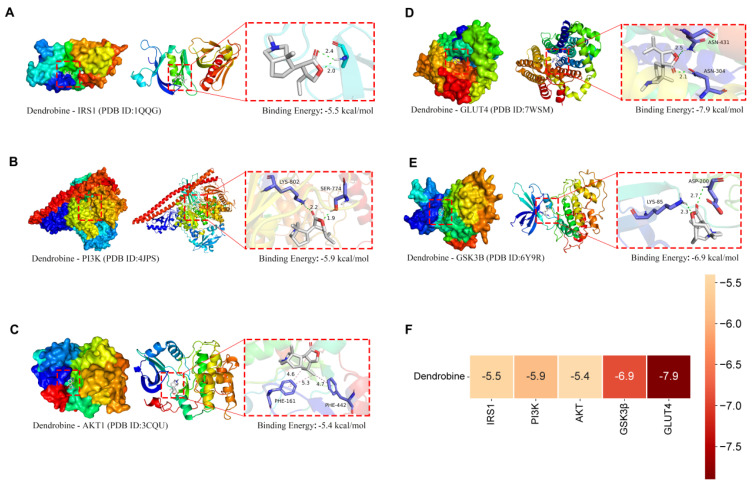
Molecular docking analysis. (**A**) The global diagrams and the details and the 2D diagrams of the docking between dendrobine and IRS1, dendrobine and PI3K (**B**), dendrobine and AKT (**C**), dendrobine and GLUT4 (**D**), and dendrobine and GSK3β (**E**). (**F**) Heat map of the optimal docking score between the dendrobine and proteins based on the binding energy.

Molecular docking serves as an advanced technique for predicting and analyzing the interactions and binding modes between ligands and receptors. In this study, the docking interactions between dendrobine from CM-YJ44-3 and receptor proteins, including IRS1 (PDB: 1QQG), PI3K (PDB: 4JPS), GLUT4 (PDB: 7WSM), Akt1 (PDB: 3CQU), and GSK3β (PDB: 6Y9R), were analyzed. Typically, binding energy less than −5 kal/mol indicates stable binding between receptor protein and small molecule, while binding energy less than −7 kcal/mol indicates strong binding [59,60,61]. The docking results in this study showed that dendrobine (Pub Chem CID: 442523) could bind well to the five receptor proteins, with binding energies below −5 kal/mol (Figure 8). Among them, dendrobine showed the strongest binding affinity, with GLUT4, with a binding energy of −7.9 kcal/mol. From the overall and detailed views, it was clear that dendrobine was bound to GLUT4 via four hydrogen bonds, indicating excellent hydrophilic interaction and strong binding affinity. These findings further support the hypothesis that dendrobine may exert its insulin-sensitizing effects by targeting key proteins in the insulin signaling pathway, providing molecular-level theoretical evidence for the hypoglycemic mechanism of CM-YJ44-3. However, these results are only analyzed from the perspective of molecular interactions, and the binding stability with target proteins and the actual biological activity still need to be validated through in vitro and in vivo experiments.

## 3. Discussion

The pathogenesis of T2D is complex and multifactorial. Therefore, developing novel and effective therapeutic strategies, identifying potential therapeutic targets, and exploring natural products have become important directions in T2D research. In recent years, natural products have garnered increasing attention due to their multi-target actions and low toxicity. For example, dietary fiber can reduce postprandial blood glucose by slowing down starch digestion [62], while plant-derived polyphenolic extracts can inhibit the activities of G6Pase and PEPCK, thereby regulating gluconeogenesis [63]. Although the hypoglycemic activity of plant-derived natural products has been extensively reported, the potential of secondary metabolites from plant endophytic microorganisms in the treatment of T2D remains largely unexplored.

*Pseudomonas protegens* was first discovered in tobacco plants in 2011, and studies have shown that it can be used as a beneficial soil bacterium to control plant diseases [64]. CM-YJ44 is a strain of *P. protegens* isolated and screened from *Dendrobium officinale*. This study is the first to explore its secondary metabolites and their hypoglycemic activity. The results showed that the third fraction of the CM-YJ44 fermentation broth (CM-YJ44-3) was able to enhance glucose uptake and glycogen synthesis, while upregulating the activities of key glycolytic enzymes, PK and HK. These findings are consistent with previous reports that phenolic acid [65] and quinoa [66] can reverse the dysregulation of HK, PK, and glucose-6-phosphatase under IR conditions, suggesting the potential of CM-YJ44-3 in regulating glucose metabolism.

To further clarify the molecular mechanism, the regulatory impact of CM-YJ44-3 on the classical insulin signaling pathway PI3K/AKT was examined. This pathway has been reported as being a pivotal regulator in insulin signal transduction in vivo. The binding of insulin to its receptor on the cell membrane activates the PI3K/AKT cascade under normal physiological conditions, subsequently driving multiple downstream metabolic reactions. The activity of this pathway is regulated by insulin sensitivity and the dynamic balance between kinases and phosphatases [67]. This study demonstrated that CM-YJ44-3 could significantly promote the phosphorylation of IRS1, thereby activating the PI3K/AKT signaling pathway. Such activation could restore insulin signaling capacity, enhance the expression and membrane translocation of GLUT4, and ultimately facilitate glucose uptake. Moreover, this pathway was also found to stimulate glycogen synthesis by promoting the expression of p-GSK3β, providing further evidence of its role in metabolic regulation. It is noteworthy that the development of T2D is often accompanied by a vicious cycle of oxidative stress and chronic inflammation [68]. In this study, ROS and NO, as representative markers of oxidative stress and inflammation, were quantitatively measured. The results revealed that CM-YJ44-3 significantly reduced ROS and NO levels, suppressed the expression of pro-inflammatory cytokines (IL-6 and TNF-α), and increased the expression of the anti-inflammatory cytokine IL-10, indicating its dual antioxidant and anti-inflammatory capabilities. These effects complement the activation of the PI3K/AKT pathway.

Given the promising hypoglycemic activity of CM-YJ44-3, its metabolite composition was further analyzed. A total of 24 metabolites were preliminarily identified in CM-YJ44-3, among which dendrobine was quantified at 79.83 ± 4.92 ng/mL. Molecular docking analysis revealed that dendrobine was capable of stably binding to five key receptor proteins within the PI3K/AKT signaling pathway, with the strongest binding affinity observed for GLUT4 (binding energy: −7.9 kcal/mol), suggesting that dendrobine may be one of the main bioactive components of CM-YJ44-3. Previous studies have reported that *Dendrobium*-derived polysaccharides [69] and polyphenols [13] improve hyperglycemia in T2D mice by modulating gut microbiota and inflammatory responses. However, similar hypoglycemic activity of dendrobine has not yet been reported. Additionally, Surendra et al. isolated a dendrobine-producing endophytic fungus, *Trichoderma longibrachiatum* MD33, from *Dendrobium officinale* rhizomes, but its bioactivity was not further explored [23]. Similarly, Zhao et al. identified an antioxidant compound, luteolin, from the metabolites of an endophytic fungus MD-R-1 isolated from Cajanus cajan, supporting the functional investigation of secondary metabolites derived from endophytic fungi [21]. It is worth noting that the endophytic fungus *Taxomyces andreanae*, isolated from *Taxus* (yew), has been confirmed to synthesize the anticancer drug paclitaxel, which has been successfully applied in clinical practice [70]. These findings underscore the pharmacological potential of microbial natural products and provide a feasible strategy to address the challenges of resource scarcity and high production costs associated with traditional plant extraction methods.

This study provides the first evidence of the hypoglycemic activity of the endophytic strain CM-YJ44, isolated from *Dendrobium officinale*, and dendrobine was preliminarily identified as one of the main components of its fermentation products. This offers a novel approach for the discovery of natural hypoglycemic agents. As a sustainable microbial resource, CM-YJ44 exhibits the characteristic of a stable and cost-effective source for dendrobine biosynthesis, offering new prospects for the screening and development of anti-hyperglycemic lead compounds. Although this study demonstrated the potential of CM-YJ44 in improving IR, the findings were primarily derived from in vitro experiments. Further evaluation in animal models and controlled clinical trials is required to assess its hypoglycemic efficacy and safety. Meanwhile, the standardization of extraction protocols and the quality control of metabolites in strain CM-YJ44 should be prioritized in future research.

## 4. Materials and Methods

### 4.1. Materials and Reagents

CM-YJ44 *(Pseudomonas protegens* CHA0, similarity 99.24%, CMCC No. 23981) was stored in the laboratory. Dendrobine (purity ≥ 98%) was purchased from Shanghai Yuanye Bio-Technology Co., Ltd. (Shanghai, China). MET was obtained from Beijing Solarbio Science and Technology Co., Ltd. (Beijing, China). Palmitic acid (PA) was obtained from Kunchuang Biotechnology Co., Ltd. (Xi’an, China). High-glucose Dulbecco’s modified Eagle’s medium (DMEM) was obtained from Gibco Co., Ltd. (Waltham, MA, USA). The complete medium for HepG2 was obtained from Wuhan Pricella Biotechnology Co., Ltd. (Wuhan, China). The primary antibodies for p-IRS1, IRS1, p-PI3K, PI3K, GLUT4, Akt, p-Akt, p-GSK3β, GSK3β, and β-actin, and secondary antibody were purchased from Proteintech Group (Wuhan, China). Acetonitrile, Methano (mass spectrometry grade) and other reagents (analytical grade) were acquired from Aladdin Co., Ltd. (Shanghai, China).

### 4.2. Preparation of CM-YJ44 Fermentation Broth

The fermentation broth and dried extract of CM-YJ44 were prepared according to the method established by our laboratory [71]. Subsequently, different fractions of CM-YJ44 were collected using the 1260 Infinity II Pre-HPLC system (Agilent, Santa Clara, CA, USA) equipped with an Eclipse Plus 95 Å C18 (4.6 × 250 mm, 5 µm) column. The specific procedure was as follows: a pure water solution was used as mobile phase A, and methanol as mobile phase B, with 10–90% B at 0–30 min. Fraction 1 (CM-YJ44-1), fraction 2 (CM-YJ44-2), and fraction 3 (CM-YJ44-3) were collected at 1–10 min, 11–20 min, and 21–30 min, respectively. The dried extracts of fractions 1–3 were prepared in the same way as the dried extracts of CM-YJ44 were prepared and then separately dissolved in methanol and dimethyl sulfoxide for experiments.

### 4.3. Cell Culture

HepG2 cells were purchased from Wuhan Pricella Biotechnology Co., Ltd. (Wuhan, China) and maintained in DMEM supplemented with 1% penicillin–streptomycin and 10% FBS at 37 °C in a humidified atmosphere containing 5% CO_2_. The cells were passaged once they reached approximately 80% confluence.

### 4.4. Cell Viability

The effect of PA, CM-YJ44, CM-YJ44-1, CM-YJ44-2, and CM-YJ44-3 on cell viability was detected by the CCK-8 assay kits (Beijing Solarbio Science and Technology Co., Ltd., China). HepG2 cells were seeded in 96-well plates at a density of 1 × 10^4^ cells/well and cultured until fully adhered. Subsequently, the medium was replaced with fresh medium containing different concentrations of PA (100–600 µmol/L) or varying concentrations of CM-YJ44-1 (100–600 ng/mL), CM-YJ44-2 (100–600 ng/mL), and CM-YJ44-3 (100–600 ng/mL). The absorbance was measured at 450 nm by a microplate reader (Bio-Rad Model 680, Bio-Rad Laboratories Ltd., Hercules, CA, USA) after 24 and 48 h of incubation. Cell viability was determined using the following formula:

$$\mathrm{cell}\;\mathrm{viability}\;\left(\%\right)=\frac{A_{1}-A_{0}}{A_{2}-A_{0}}\times 100\%$$

where *A*_1_, *A*_2_, and *A*_0_ are the absorbances of the sample group (PA, CM-YJ44-1, CM-YJ44-2, and CM-YJ44-3 treatment), control group (untreated cells), and blank group (only medium), respectively.

### 4.5. Establishment of the IR Model and Glucose Consumption of Samples on the IR HepG2 Cell

The construction of the IR model followed the method based on the previous studies [12]. HepG2 cells were seeded onto 96-well plates at a density of 1 × 10^6^ cells/well. Following a medium change, each well received fresh medium containing PA at concentrations ranging from 100 to 400 µmol/L. After incubation periods of 12, 24, 36, or 48 h, glucose levels in the supernatant were assessed using a glucose assay kit (Nanjing Jiancheng Bioengineering Institute, Nanjing, China), and the most appropriate PA concentration for model induction was determined.

Following the procedures described above, once the cell model was successfully established, MET (300 ng/mL) and varying concentrations of the samples (CM-YJ44-3-L: 100 ng/mL, CM-YJ44-3-M: 200 ng/mL, and CM-YJ44-3-H: 300 ng/mL) were individually added to each well and incubated for 36 h to assess glucose consumption.

### 4.6. Determination of Intracellular Glycogen, HK, and PK

After the cell model was established, MET and various concentrations of CM-YJ44-3 were added for a 36-hour incubation. Then, the cells were harvested through trypsin digestion and centrifugation. The intracellular glycogen content, as well as PK and HK activities, were then determined using the respective assay kits for glycogen, HK, and PK (Beijing Solarbio Science and Technology Co., Ltd., China) in accordance with the manufacturer’s protocols.

### 4.7. Determination of Intracellular ROS Levels

IR cell model establishment and sample administration were consistent with 2.5. According to the ROS kit instructions (Beijing Solarbio Science and Technology Co., Ltd., China), 1 mL of 2,7-Dichlorodihydrofluorescein diacetate (DCFH-DA) solution at a concentration of 10 µmol/mL was applied. The cells were incubated at 37 °C for 30 min. Cell morphology was observed using fluorescence microscopy, and the results were analyzed with Image-Pro Premier 6.0.

### 4.8. Levels of Determination of Intracellular NO

The IR cell model was established, and samples were administered following the method described in Section 2.5. Subsequently, the cells were collected after trypsin digestion and centrifugation. The NO content was measured using the NO assay kits (Beijing Solarbio Science and Technology Co., Ltd., China) according to the manufacturer’s instructions.

### 4.9. Metabolites Identification of CM-YJ44-3

#### 4.9.1. Qualitative Analysis of CM-YJ44-3 Using the QE

The CM-YJ44-3 sample was analyzed using a Q Exactive Focus LC-MS System (Thermo, Waltham, MA, USA) coupled with a Waters ACQUITY UPLC BEH C18 column (1.8 µm, 2.1 × 100 mm). The HPLC conditions were as follows: the column temperature was set at 30 °C; mobile phase consisted of 0.1% formic acid in water (A) and 0.1% formic acid in acetonitrile (B), and the linear gradient was as follows: 95–10% A at 0–20 min, 10–95% A at 20–30 min; and the flow rate was 0.3 mL/min, and the injection volume was 0.5 µL. Mass spectrometry analysis was performed using a QE mass spectrometer (Thermo, Waltham, MA, USA) equipped with an electrospray ionization (ESI) source. Full MS scans were conducted in polarity switching mode with a resolution of 70,000 (defined at m/z 200) and a scan range of 100 to 1500 m/z. The maximum ion injection time was set to 50 ms, and the automatic gain control target was set to 1e5. For data-dependent MS/MS, the resolution was 17,500, with an isolation window of 3.0 m/z and stepped normalized collision energies (NCE) of 15, 30, and 45. Dynamic exclusion was set to 8.0 s to prevent repeated analysis of the same ions, and the apex trigger time was set between 2 and 6 s to ensure MS/MS scans were acquired at the chromatographic peak apex. Subsequently, qualitative analysis of metabolites was performed using Compound Discoverer 2.1 and the MzCloud database, based on ionization mode, fragment ion m/z, abundance, and retention time.

#### 4.9.2. Quantitative Analysis of CM-YJ44-3 Using UPLC-MS/MS

A SCIEX Triple Quad 5500+ system (SCIEX, Framingham, MA, USA) equipped with a Waters ACQUITY UPLC BEH C18 (1.7 µm, 2.1 × 50 mm) column was applied to analyze the CM-YJ44-3 sample. The HPLC conditions were as follows: The column temperature was set at 40 °C. The mobile phase consisted of acetonitrile (A) and 0.1% formic acid in water, and the linear gradient consisted of 10–60% A at 0–5 min, 60–100% A at 5–8 min, 100% A at 8–10 min, 100–10% A at 10–10.1 min and 10% A at 10.1–13 min; the flow rate was 0.3 mL/min, and the injection volume was 0.5 µL. The parameters for LC-MS analysis were as follows: equipped with an electrospray ionization and Multiple Reaction Monitoring, positive and negative ion scanning was used according to the metabolites; gas pressure was 30 psi; nebulizer gas and heater gas were set to 50 psi, respectively; collision gas was set to medium; and ionic voltage was 5500 V/−4500 V. Ion pairs were listed in Table S1.

### 4.10. Western Blot Assay

After MET and CM-YJ44-3 treatments, the cells were washed and lysed using RIPA lysis buffer (Beyotime, Shanghai, China). The supernatant was collected after centrifugation (12,000× *g*, 15 min, 4 °C), and protein concentration was quantified using a BCA kit (Applygen, Beijing, China). Proteins (30 μg) were separated by 8% SDS-PAGE (5% stacking gel) and transferred onto 0.45 μm PVDF membranes (Millipore, Burlington, MA, USA) at 300 mA constant current for 60 min. After blocking with 5% BSA-TBST for 1 h at room temperature, membranes were incubated with primary antibodies (1:1000 in 5% BSA-TBST) at 4 °C overnight, followed by three 10-minute TBST washes. Then, the membranes were incubated with HRP-conjugated goat anti-rabbit/mouse IgG (H + L) (1:10,000 in 5% BSA-TBST) for 1 h at room temperature and washed again with TBST (3 × 10 min). Protein signals were detected using Super ECL Plus (Millipore, MA, USA) with 3–5 min reaction time and visualized using an ESSENTIAL V6 system (UVITEC, Cambridge, UK), with exposure times ranging from 10 s to 5 min. β-Actin was used as the internal control.

### 4.11. RNA Isolation and RT-PCR

The IR cell model establishment and sample administration were consistent with 2.5. The total mRNA was extracted from cultured cells using the mRNA extraction kit (Aidlab Biotechnologies Co., Ltd., Beijing, China) and transcribed into cDNA using the PrimeScript RT reagent Kit (Aidlab Biotechnologies Co., Ltd., China). The cDNAs were then quantified by SYBR Premix Ex Taq (Novoprotein, Suzhou, China) on the ABI 7500 Fast Real-Time PCR System (Applied Biosystems, Waltham, MA, USA). The sequences for the RT-PCR primers are listed in Supplementary Table S2.

### 4.12. Molecular Docking

Protein structures were obtained from the Protein Data Bank (PDB) database, including AKT1 (PDB ID: 3CQU), GLUT4 (PDB ID: 7WSM), GSK3β (PDB ID: 6Y9R), IRS1 (PDB ID: 1QQG), and PI3K (PDB ID: 4JPS). The protein structures were pre-processed using PyMOL 2.5.2 to remove crystal waters, ions, and non-relevant ligands. Polar hydrogen atoms were added, and Gasteiger charges were calculated using AutoDock Tools 1.5.7, and the processed structures were saved in .pdbqt format. The 3D structure of the small-molecule dendrobine (PubChem CID: 442523) was downloaded in .sdf format and converted to .pdb format using OpenBabel 3.1.1, followed by energy minimization to ensure structural stability. Hydrogen atoms and charges were subsequently added using AutoDock Tools, and the structure was saved in .pdbqt format for molecular docking.

The docking target regions were defined based on the literature and known protein active sites, and grid parameters were set as shown in Table 3. Docking calculations were performed using AutoDock Vina 1.2.0, with default settings. Binding affinities and ligand–receptor binding conformations were recorded.

To validate the reliability of the docking method, the co-crystallized ligand was used as a positive control. The root-mean-square deviation (RMSD) between the original co-crystal ligand and its re-docked conformation was calculated. An RMSD value of less than 2 Å was considered indicative of successful methodological validation [72]. Detailed validation results are shown in Supplementary Figure S1.

### 4.13. Statistical Analysis

All experimental data are expressed as the mean ± SD (*n* = 3). The gray values from Western blot assays and ROS analysis were analyzed using Image-Pro Premier 6.0 (Media Cybernetics, Rockville, MD, USA). One-way ANOVA followed by Tukey’ s post hoc test (LSR) was performed using IBM SPSS Statistics 27.0 (IBM Corp., Armonk, NY, USA). Graphs and data visualizations were created with GraphPad Prism 9.5.1 (GraphPad Software, San Diego, CA, USA). *p* < 0.05 was considered statistically significant.

## 5. Conclusions

In summary, this study systematically evaluated the activities of the secondary metabolites (CM-YJ44-3) of the endophytic bacterium CM-YJ44 (*Pseudomonas protegens* CHA0, 99.24%) from *Dendrobium officinale* on the improvement of IR. The results revealed that CM-YJ44-3 could significantly promote glucose uptake and glycogen synthesis, enhance the activities of PK and HK, and restore insulin signaling by activating the IRS1/PI3K/AKT/GSK3β/GLUT4 pathway. In addition, CM-YJ44-3 significantly suppressed the production of ROS and NO, downregulated pro-inflammatory cytokines, and upregulated anti-inflammatory cytokines, ultimately alleviating IR.

A total of 24 metabolites were preliminarily identified in CM-YJ44-3, and dendrobine was the major component, with a concentration of 78.93 ± 4.29 ng/mL. Molecular docking analysis revealed that dendrobine exhibited strong binding affinities with two key functional proteins, namely GLUT4 and GSK3β, with binding energies of −6.9 kcal/mol and −7.9 kcal/mol, respectively, suggesting that dendrobine may be one of the critical metabolites mediating the bioactivity of CM-YJ44-3. As a sustainable microbial resource, the strain CM-YJ44 represents a sustainable biotechnological resource with potential for industrial-scale production through fermentation and genetic engineering, positioning it as a viable candidate for hypoglycemic agents or functional food additives. Nevertheless, this study was primarily conducted in vitro; the mechanisms and toxicological profiles require further validation through in vivo animal models and controlled clinical trials. Future studies should focus on the purification and toxicological evaluation of CM-YJ44-derived metabolites to facilitate the application in the prevention and treatment of metabolic diseases such as T2D.

## Figures and Tables

**Table 1:** Identification of components of CM-YJ44-3.

Components	Molecular Formula	Molecular Weight	[M + H]+	[M − H]−	Retention Time (S)	Classification	Know Sources
Embelin *	C17H26O4	294.18311	295.19039	293.17583	10.19	Benzoquinone derivative	Plant [25]
Protopine *	C20H19NO5	353.12632	354.1336	352.11905	12.25	Alkaloids	Plant [26]
Dendrobine	C16H25NO2	263.27234	262.18125	264.19581	4.72	Alkaloids	Fungi [27], plant [28]
7-Hydroxycoumarin *	C9H6O3	162.03169	163.03897	161.02442	20.05	Coumarins	Plant [29]
Xanthohumol *	C21H22O5	354.42452	353.13945	355.154	8.91	Flavonoids	Plant [30]
Rutin	C27H30O16	610.51842	609.14611	611.16066	12.70	Flavonoids	Fungi [31], plant [32]
Chrysin	C15H10O4	254.24234	253.05063	255.06519	6.72	Flavonoids	Fungi [33], plant [34]
Scopoletin	C10H8O4	192.17255	191.03498	193.04954	8.35	Coumarins	Fungi [35], plant [36]
Nardosinone *	C15H22O3	250.15689	249.14962	251.16417	11.73	Sesquiterpenoids	Plant [37]
Azelaic acid	C9H16O4	188.10486	189.11214	187.09758	5.90	Organic acids	Fungi [38], plant [39]
Benzoic acid	C7H6O2	122.03678	123.04406	121.0295	13.46	Organic acids	Bacteria [40], plant [41]
Caffeic acid	C9H8O4	180.04226	181.04954	179.03498	5.65	Phenolic compounds	Bacteria [42], plant [43]
Cinnamic acid	C9H8O2	148.05243	137.05971	135.04515	18.84	Phenolic compounds	Fungi [44], plant [45]
20 (R)-Ginsenoside Rh1	C36H62O9	638.43938	639.44666	637.43211	5.50	Saponins	Fungi [46], plant [47]
Ginsenoside F1	C36H62O9	638.43938	639.44666	637.43211	5.68	Saponins	Fungi [46], plant [47]
Ginsenoside F2	C42H72O13	784.49729	785.50457	783.49002	10.54	Saponins	Fungi [48], plant [47]
Ginsenoside Rg2	C42H72O13	784.49729	785.50457	783.49002	10.54	Saponins	Fungi [48], plant [47]
Atractylodin	C13H10O	182.07316	183.08044	181.06589	11.06	Terpenes	Fungi [49], plant [50]
Gingerol	C17H26O4	294.18311	295.19039	293.17583	10.19	Terpenes	Fungi [51], plant [52]
Artemisinic acid *	C15H22O2	234.16198	235.16926	233.1547	13.08	Terpenes	Plant [53]
Pinoresinol	C22H26O6	386.17294	387.18022	385.16566	9.96	Terpenes	Fungi [54], plant [55]
Eudesmin *	C22H26O6	386.17294	355.19039	353.17583	7.92	Terpenes	Plant [56]
Curcumene	C15H22O2	234.16198	235.16926	233.1547	13.08	Terpenes	Fungi [57], plant [58]
Benzyl glycolate *	C9H10O3	166.06299	167.07027	165.05572	5.11	Miscellaneous Compounds	Synthetic compound

* Metabolites detected for the first time in *Pseudomonas*.

**Table 2:** Content of dendrobine metabolites of CM-YJ44-3.

Constituents	Stand Cure Equation	R^2^	Concentration Range (ng/mL)	Content (ng/mL)
Dendrobine	y = 6553.47949x − 268.84194	0.9980	0.5–200	78.63 ± 4.29

**Table 3:** Grid box center coordinates and dimensions used for docking dendrobine to target proteins.

Protein	PDB ID	Center (X, Y, Z)	Size (X × Y × Z)
AKT1	3CQU	6, 0, 19	26 × 26 × 24
GLUT4	7WSM	101, 103, 108	37 × 29 × 29
GSK3B	6Y9R	−14, −14, −5	22 × 22 × 20
IRS1	1QQG	8, 48, 36	44 × 58 × 66
PI3K	4JPS	−11, −4, 16	33 × 20 × 27

## Data Availability

The original contributions presented in this study are included in the article/Supplementary Materials. Further inquiries can be directed to the corresponding authors.

## Supplementary Materials

The following supporting information can be downloaded at https://www.mdpi.com/article/10.3390/ph18060817/s1, Figure S1: Results of validation. Figure S2: Total ion chromatogram. (A) CM-YJ44-3. (B) Blank control. Figure S3: Standard curve of dendrobine; Figure S4. Calibration curve and chromatogram of CM-YJ44-3 in dendrobine quantitative analysis; Table S1: Ion pair informations. Table S2: Target genes and primers for RT-PCR. Table S3: Calibration curve and CM-YJ44-3 concentration table for dendrobine quantification.
