# Correction: Qin et al. Metabolites from the *Dendrobium* Endophyte *Pseudomonas protegens* CM-YJ44 Alleviate Insulin Resistance in HepG2 Cells via the IRS1/PI3K/Akt/GSK3β/GLUT4 Pathway. *Pharmaceuticals* 2025, *18*, 817

**DOI:** 10.3390/ph18081234

**Published:** 2025-08-21

**Authors:** Luqi Qin, Yixia Zhou, Bei Fan, Jiahuan Zheng, Rao Diao, Jiameng Liu, Fengzhong Wang

**Affiliations:** 1 Key Laboratory of Agro-Products Quality and Safety Control in Storage and Transport Process, Ministry of Agriculture and Rural Affairs, Institute of Food Science and Technology, Chinese Academy of Agricultural Sciences, No. 2, Yuanmingyuan West Road, Haidian District, Beijing 100193, China; qlq120011@163.com (L.Q.);; 2 National Nanfan Research Institute (Sanya), Chinese Academy of Agricultural Sciences, Sanya 572024, China

## Error in Table

In the original publication [1], there were some mistakes in Table 1 as published. In Table 1, the following corrections should be made: Concerning the compound Dendrobine, the values of M+H^+^ and M−H^−^ were mistakenly reversed; specifically, the correct M+H^+^ value should be 264.19581, and the correct M−H^−^ value should be 262.18125. Concerning the compound Xanthohumol, the values of M+H^+^ and M−H^−^ were mistakenly reversed; the correct M+H^+^ value should be 355.154, the correct M−H^−^ value should be 353.13945, and the correct classification should be Coumarins. Concerning the compound Rutin, the values of M+H^+^ and M−H^−^ were mistakenly reversed; the correct M+H^+^ value should be 611.16066, and the correct M−H^−^ value should be 609.14611. Concerning the compound Chrysin, the values of M+H^+^ and M−H^−^ were mistakenly reversed; the correct M+H^+^ value should be 255.06519, and the correct M−H^−^ value should be 253.05063. Concerning the compound Scopoletin, the values of M+H^+^ and M−H^−^ were mistakenly reversed; the correct M+H^+^ value should be 193.04954, the correct M−H^−^ value should be 191.03498, and the correct classification should be Flavonoids. Concerning the compound Nardosinone, the correct classification should be Miscellaneous Compounds. Concerning the compound Ginsenoside F2, the correct retention time should be 12.87. Concerning the compound Pinoresinol, the values of M+H^+^ and M−H^−^ were incorrectly presented; the “Molecular Formula” should be C_20_H_26_O_6_, and the “Molecular Weight” value should be 354.17801; the correct M+H^+^ value should be 355.19039, and the correct M−H^−^ value should be 353.17583. Concerning the compound Chrysin, the values of M+H^+^ and M−H^−^ were incorrectly presented; the correct M+H^+^ value should be 387.18022, and the correct M−H^−^ value should be 385.16566.

The corrected legend appears below:

**Table 1:** Identification of components of CM-YJ44-3.

Components	Molecular Formula	Molecular Weight	[M+H]^+^	[M−H]^−^	Retention Time (S)	Classification	Known Sources
Embelin *	C_17_H_26_O_4_	294.18311	295.19039	293.17583	10.19	Benzoquinone derivative	Plant [25]
Protopine *	C_20_H_19_NO_5_	353.12632	354.1336	352.11905	12.25	Alkaloids	Plant [26]
Dendrobine	C_16_H_25_NO_2_	263.27234	264.19581	262.18125	4.72	Alkaloids	Fungi [27], Plant [28]
7-Hydroxycoumarin *	C_9_H_6_O_3_	162.03169	163.03897	161.02442	20.05	Coumarins	Plant [29]
Xanthohumol *	C_21_H_22_O_5_	354.42452	355.154	353.13945	8.91	Coumarins	Plant [30]
Rutin	C_27_H_30_O_16_	610.51842	611.16066	609.14611	12.7	Flavonoids	Fungi [31], Plant [32]
Chrysin	C_15_H_10_O_4_	254.24234	255.06519	253.05063	6.72	Flavonoids	Fungi [33], Plant [34]
Scopoletin	C_10_H_8_O_4_	192.17255	193.04954	191.03498	8.35	Flavonoids	Fungi [35], Plant [36]
Nardosinone *	C_15_H_22_O_3_	250.15689	249.14962	251.16417	11.73	Miscellaneous Compounds	Plant [37]
Azelaic acid	C_9_H_16_O_4_	188.10486	189.11214	187.09758	5.9	Organic Acids	Fungi [38], Plant [39]
Benzoic acid	C_7_H_6_O_2_	122.03678	123.04406	121.0295	13.46	Organic Acids	Bacteria [40], Plant [41]
Caffeic acid	C_9_H_8_O_4_	180.04226	181.04954	179.03498	5.65	Phenolic Compounds	Bacteria [42], Plant [43]
Cinnamic acid	C_9_H_8_O_2_	148.05243	137.05971	135.04515	18.84	Phenolic Compounds	Fungi [44], Plant [45]
20 (R)-Ginsenoside Rh1	C_36_H_62_O_9_	638.43938	639.44666	637.43211	5.5	Saponins	Fungi [46], Plant [47]
Ginsenoside F1	C_36_H_62_O_9_	638.43938	639.44666	637.43211	5.68	Saponins	Fungi [46], Plant [47]
Ginsenoside F2	C_42_H_72_O_13_	784.49729	785.50457	783.49002	12.87	Saponins	Fungi [48], Plant [47]
Ginsenoside Rg2	C_42_H_72_O_13_	784.49729	785.50457	783.49002	10.54	Saponins	Fungi [48], Plant [47]
Atractylodin	C_13_H_10_O	182.07316	183.08044	181.06589	11.06	Terpenes	Fungi [49], Plant [50]
Gingerol	C_17_H_26_O_4_	294.18311	295.19039	293.17583	10.19	Terpenes	Fungi [51], Plant [52]
Artemisinic acid *	C_15_H_22_O_2_	234.16198	235.16926	233.1547	13.08	Terpenes	Plant [53]
Pinoresinol	C_20_H_26_O_6_	354.17801	355.19039	353.17583	9.96	Terpenes	Fungi [54], Plant [55]
Eudesmin *	C_22_H_26_O_6_	386.17294	387.18022	385.16566	7.92	Terpenes	Plant [56]
Curcumene	C_15_H_22_O_2_	234.16198	235.16926	233.1547	13.08	Terpenes	Fungi [57], Plant [58]
Benzyl glycolate *	C_9_H_10_O_3_	166.06299	167.07027	165.05572	5.11	Miscellaneous Compounds	Synthetic compound

* Metabolites detected for the first time in *Pseudomonas*.

The authors state that the scientific conclusions are unaffected. This correction was approved by the Academic Editor. The original publication has also been updated.
