# Supplementary material for: Metabolites from the Dendrobium Endophyte Pseudomonas protegens CM-YJ44 Alleviate Insulin Resistance in HepG2 Cells via the IRS1/PI3K/Akt/GSK3β/GLUT4 Pathway
Source: Pharmaceuticals (Basel). 2025 May 29;18(6):817. doi: 10.3390/ph18060817 (PMC12196277; doi:10.3390/ph18060817)
Supplement: Supplementary file 1 [file pharmaceuticals-18-00817-s001.zip › pharmaceuticals-3624697-supplementary.pdf]

Supporting Information

Table S1. Ion pair informations

Component	Q1 Mass（Da）	Q3 Mass （Da）	DP（V）	CE(V)
Dendrobine	264.2	105.2	140	50

Metabolites from the *Dendrobium* Endophyte Pseudomonas protegens CM-YJ44 Alleviate Insulin Resistance in HepG2 Cells via the IRS1/PI3K/Akt/GSK3β/GLUT4 Pathway

Luqi Qin[1], Yixia Zhou[1], Bei Fan[1], Jiahuan Zheng[1], Rao Diao[1], Jiameng Liu[1,2,*] and Fengzhong Wang[1,*]

[1] Key Laboratory of Agro-Products Quality and Safety Control in Storage and Transport Process, Ministry of Agriculture and Rural Affairs, Institute of Food Science and Technology, Chinese Academy of Agricultural Sciences, No. 2, Yuanmingyuan West Road, Haidian District, Beijing 100193, China;

qlq120011@163.com (L.Q.); zyx13967696166@163.com (Y.Z.); fanbei@caas.cn (B.F.);

zjiah1996@163.com (J.Z.); diaodiao19971126@sina.com (R.D.)

[2] National Nanfan Research Institute (Sanya), Chinese Academy of Agricultural Sciences, Sanya 572024, China

[*]Correspondence: liujiameng@caas.cn (J.L.), Tel./Fax: +86-1062815969; wangfengzhong@caas.cn (F.W.); Tel./Fax: +86-1062815969

1. UPLC-MS/MS Analysis of CM-YJ44-3

2. RNA isolation and RT-PCR

Table S2. Target genes and primers for RT - PCR

Gene	Forward primer (5’- 3’)	Reverse primer (5’- 3’)
β-actin	TGTCCACCTTCCAGCAGATGT	AGCTCAGTAACAGTCCGCCTAGA
TNF-α	AGGACCAGCTAAGAGGGAGA	TTCAGTGCTCATGGTGTCCT
IL-8	TGGCAGCCTTCCTGATTTCT	AATTTCTGTGTTGGCGCAGT
IL-10	AGGGCACCCAGTCTGAGAACA	AGGGCACCCAGTCTGAGAACA

3. Validation of the molecular docking methodology

To validate the molecular docking methodology used in this study, GLUT4 protein was selected as a representative example. The co-crystallized ligand Cytochalasin B (PubChem CID: 5311281) was extracted from the GLUT4 structure using PyMOL. The ligand was then re-docked into the binding site of GLUT4

using the same docking protocol applied in this study. The root mean square deviation (RMSD) between the re-docked pose and the original co-crystallized pose was calculated. An RMSD value of less than 2 Å indicates that the docking method is reliable. The results showed that the re-docked pose of Cytochalasin B closely overlapped with its original position and orientation in the crystal structure, with an RMSD of 0.011 Å, confirming the validity of the docking methodology.

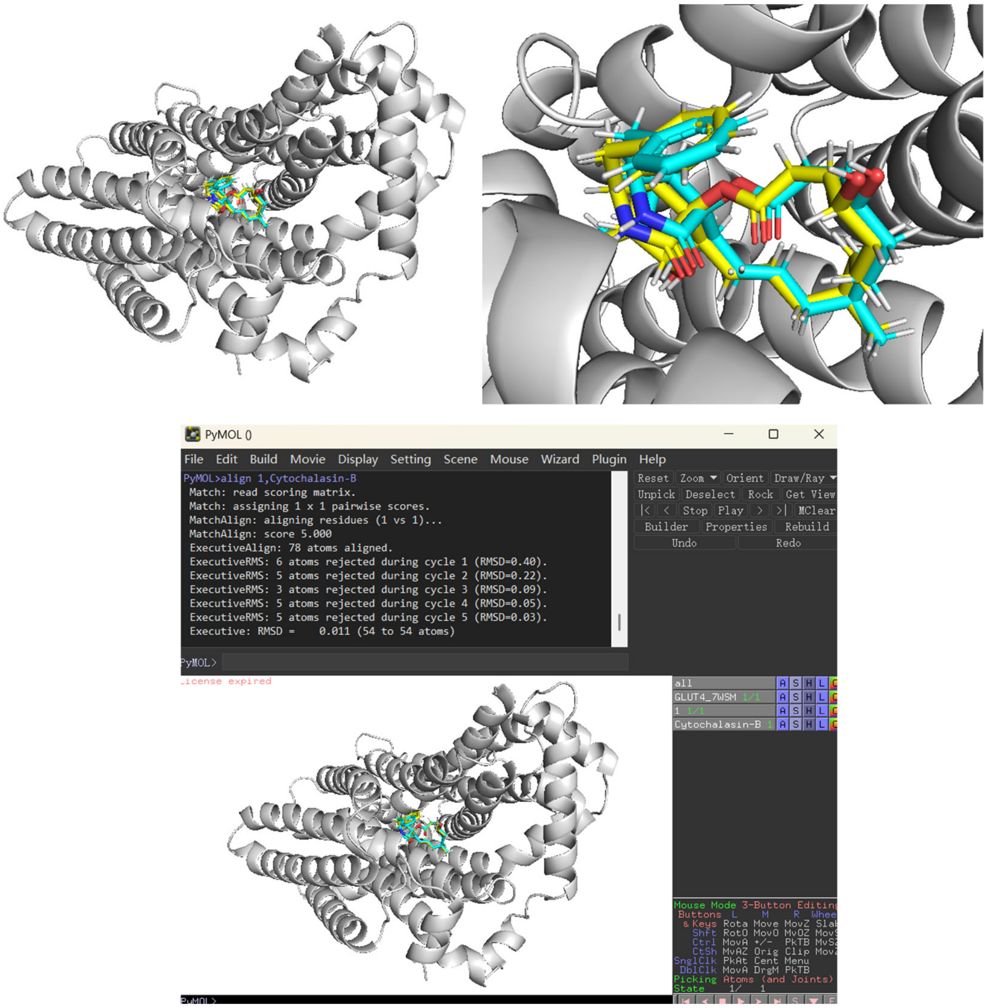

Figure S1 Results of validation

4. Components Identification of CM-YJ44-3 Using Q Exactive Focus LC-MS System

A

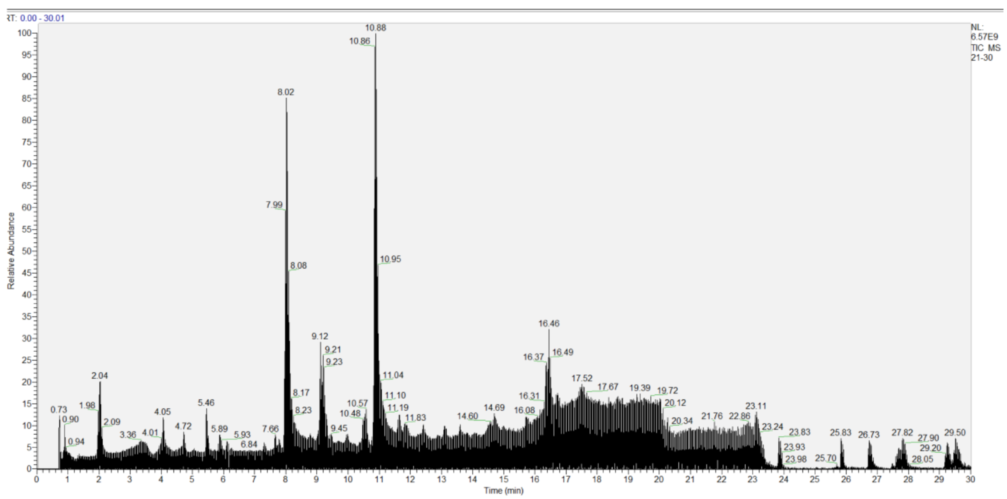

B

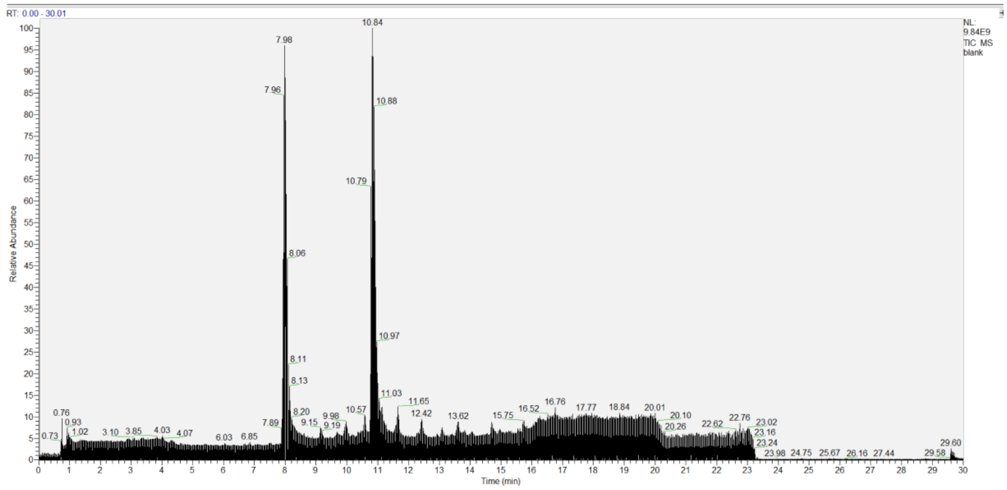

Figure S2. Total ion chromatogram. (A) CM-YJ44-3. (B) Blank control

5. UPLC-MS/MS analysis dendrobine of CM-YJ44-3

Table S3. Calibration curve and CM-YJ44-3 concentration table for dendrobine quantification

Sample Name	Sample Type	Area (cps)	RT (min)	Target [Conc]. (ng/ml)	Calculated Conc. (ng/ml)
Rstd_0.1ng	Standard	7.150e+02	3.60	0.100	0.150
Rstd_0.2ng	Standard	2.190e+03	3.60	0.200	0.375
Rstd_0.5ng	Standard	2.916e+03	3.61	0.500	0.486
Rstd_1ng	Standard	6.433e+03	3.61	1.000	1.023
Rstd_2ng	Standard	1.373e+04	3.61	2.000	2.135
Rstd_5ng	Standard	3.211e+04	3.61	5.000	4.941
Rstd_10ng	Standard	6.612e+04	3.61	10.000	10.130

Rstd_20ng	Standard	1.343e+05	3.60	20.000	20.531
Rstd_50ng	Standard	3.309e+05	3.61	50.000	50.534
Rstd_100ng	Standard	6.146e+05	3.61	100.000	93.822
Rstd_200ng	Standard	1.259e+06	3.61	200.000	192.200
CM-YJ44-3-1	Unknown	5.182e+05	3.60	N/A	79.117
CM-YJ44-3-2	Unknown	4.864e+05	3.60	N/A	74.263
CM-YJ44-3-3	Unknown	5.424e+05	3.60	N/A	82.810

$y = 6553.47949\ x\ -268.84194\ (R^2 = 0.99800)$

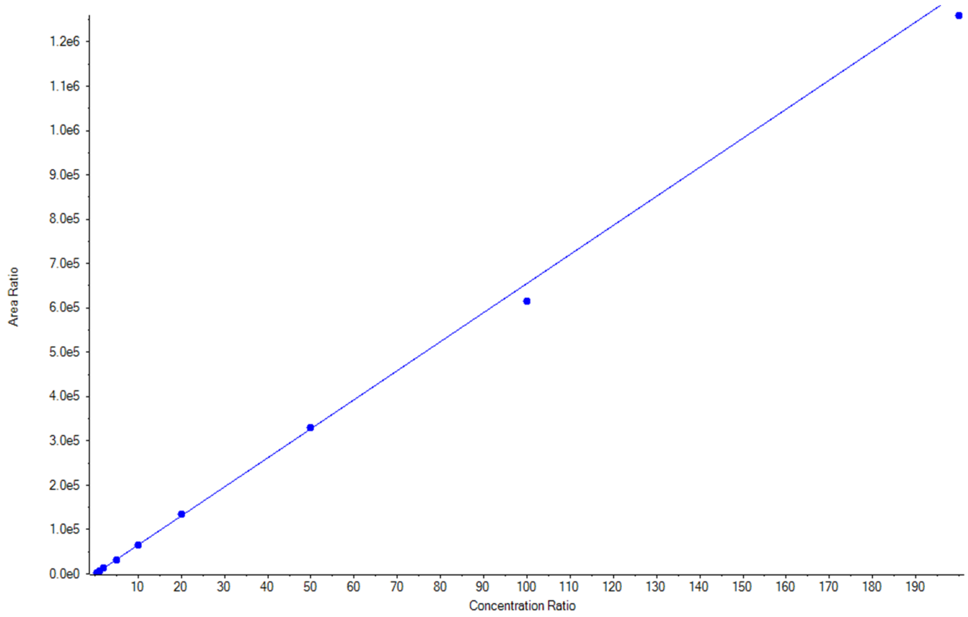

Figure S3. Standard curve of dendrobine.

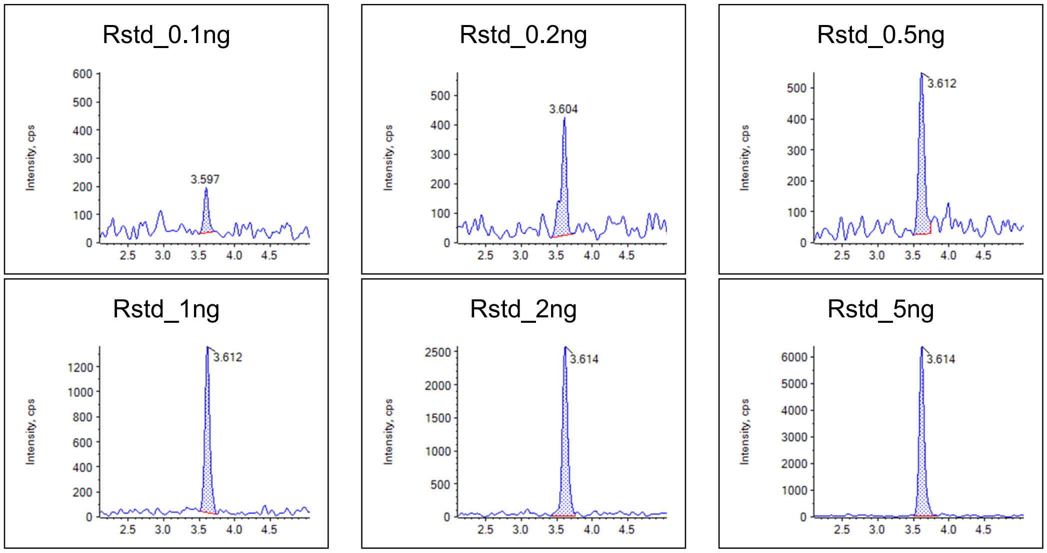

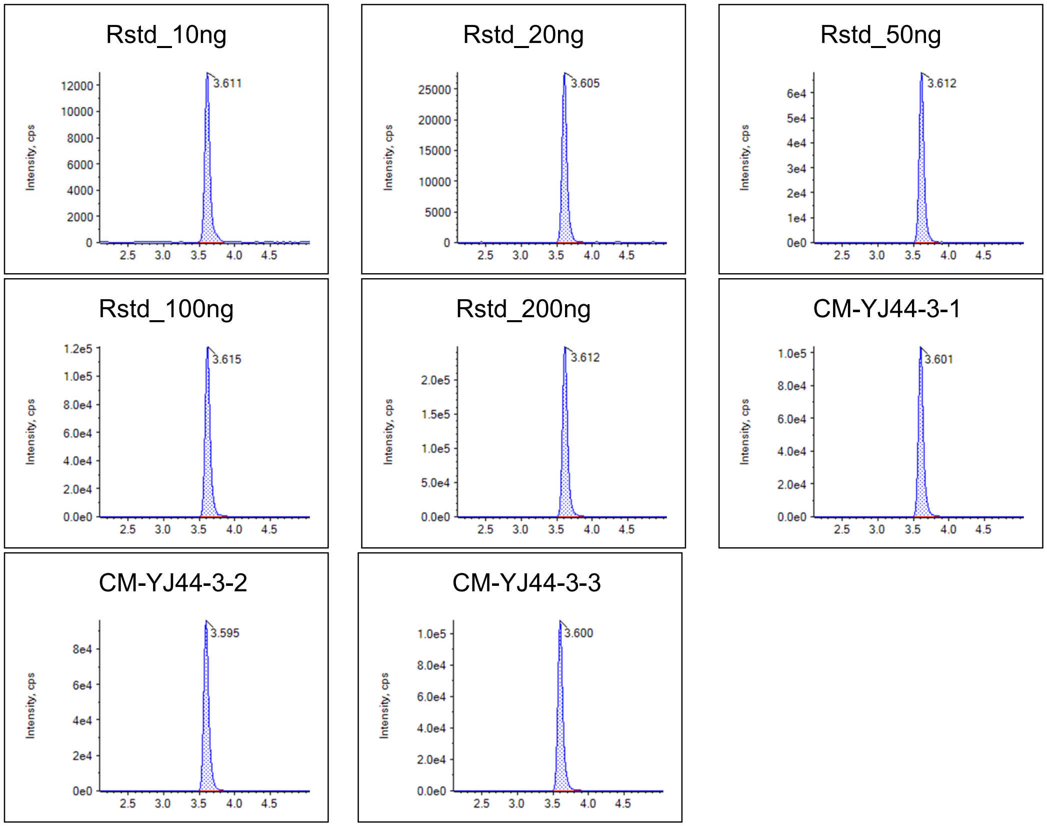

Figure S4. Calibration curve and chromatogram of CM-YJ44-3 in dendrobine quantitative analysis.
